# Human competition is not lower if competing is socially wasteful instead of socially beneficial

**DOI:** 10.1038/s41598-022-14891-7

**Published:** 2022-06-24

**Authors:** Kasper Otten

**Affiliations:** Department of Sociology, Utrecht University, Padualaan 14, 3584 CH Utrecht, The Netherlands

**Keywords:** Human behaviour, Psychology and behaviour

## Abstract

Humans compete for jobs, promotions, income, status, and many other scarce goods. In some situations, allocating scarce goods via competition is socially beneficial. In other situations, competition is not necessary to allocate goods, and nevertheless engaging in competition creates inefficiencies and welfare loss. We use an incentivized lab experiment to study whether people compete differently depending on whether allocating scarce goods via competition is socially wasteful or socially beneficial. We find that competition behavior is strikingly similar in situations where competing is socially wasteful and socially beneficial. Accordingly, there is large excess competition in situations of wasteful competition, creating considerable efficiency losses. We find evidence of a social trap involved in this excess competition. People are considerably more likely to compete if they believe others compete, and their beliefs on others’ competition are similar in situations where competing is socially wasteful and socially beneficial. Interventions aimed at lowering beliefs on others’ competition may be an effective method of lowering excess competition to prevent inefficiencies and welfare loss.

## Introduction

Competition over scarce goods is a central element of human life^1,2^. Students compete for the best grades, job seekers compete for vacancies, academics compete for grants and tenure, companies compete for market share, and so on. Sometimes allocating scarce goods via competition is socially beneficial. It can motivate students to learn more, lead to better research among academics, and push companies to produce better goods. In other instances, competition to allocate scarce goods is socially wasteful. People may compete for social status through wasteful spending on clothes, cars, and houses to “keep up with the Joneses”. Employees may compete for their employers’ approval by working over-hours and neglecting their personal lives. Academics may compete with each other in their number of publications, sometimes favoring quantity over quality. The famous analogy for situations of wasteful competition is that of a rat race, in which rats compete with each other to be the first to get to a piece of cheese even though the race produces no additional cheese. It is not uncommon for competition in the domains of education^3^, work^4^, and academia^5^ to be compared to rat races. A central feature of competition structures is thus whether competing is socially beneficial or socially wasteful. Although there is abundant research documenting both situations in which allocating scarce goods via competition is socially beneficial and socially wasteful^6–9^, we know surprisingly little about how people condition their behavior on this aspect of the competition structure. We use an incentivized lab experiment to study this issue.

Ideally, people would compete less in situations where allocation of goods via competition is socially wasteful than in situations where allocation of goods via competition is socially beneficial. However, the numerous examples of excessive and unnecessary competition in everyday life make clear that people also frequently engage in socially wasteful competitions. A desire or norm to win may drive individuals to enter competitions even if there is no actual need for competition and everybody would be better off if nobody competes^10^. At the same time, there is abundant evidence that people do care about collective efficiency and others’ welfare^11^, suggesting that they may compete less in situations where competition is wasteful and welfare-reducing. At present, we know little about how people compete differently in situations where the allocation of goods via competition is socially beneficial or socially wasteful.

Instances of beneficial and wasteful competition in society often differ not only in whether the allocation of goods via competition is beneficial or wasteful, but also in several other aspects. They may differ, among others, in terms of the population, complexity of the competition structure, and knowledge about others’ behavior. Lab experiments can help in this regard to isolate the causal effect of the wastefulness of allocating goods via competition, keeping all other factors constant. In our experiment, we let participants decide whether to enter a competition to win one of a limited number of rewards. We vary whether participants make this choice in a condition where competing is socially beneficial or in a condition where competing is socially wasteful.

The incentivized lab experiment uses a simple competition model that has been used to study micro-level decisions to compete in interdependent situations^12–15^. In the model, there are a limited number of rewards to be allocated among a group of actors. Rewards are scarce in the sense that the number of rewards is always below the number of group members. Actors can incur a cost to enter a competition to increase their chance of winning one of the rewards or they can abstain from competing. The chance of winning a reward depends on the number of rewards and how many actors enter the competition. Rewards first go to individuals that entered the competition. If the number of competitors is exactly equal to the number of rewards, all competitors receive a reward and all non-competitors do not. If the number of competitors exceeds the number of rewards, the rewards will be randomly allocated among the competitors. This means that some competitors will not get a reward despite having incurred the cost to enter the competition. Although this simple competition model does not encompass all potential forms of competition, it captures its elementary aspect as commonly defined: “competition is a rivalry between individuals (or groups or nations), and it arises whenever two or more parties strive for something that all cannot obtain”^16^.

The crucial element that we vary in two conditions of the model is what happens if there are fewer competitors than rewards, i.e., if there are excess rewards. In the first condition, we do not allocate these excess rewards. Thus, rewards will be lost if there are not enough competitors, meaning that there need to be enough competitors for a collectively efficient allocation of rewards (i.e., the allocation that leads to the highest total group payoffs). Here, competing is socially beneficial up to the point where the number of competitors equals the number of rewards. In the second condition, we allocate excess rewards among the non-competitors. This means that it is collectively efficient if nobody competes, as then nobody incurs the cost of competing and the rewards are allocated for free. This condition resembles the situations in which competition is socially wasteful; it is costly and produces no additional value. The order of individual payoffs of the model, from high to low, is: (1) winning a reward without competing, (2) winning a reward by competing, (3) not winning a reward without competing, and (4) not winning a reward despite competing. Decisions in the experiment are incentivized with monetary stakes.

We will refer to the condition where excess rewards are unallocated as the unallocated condition and the condition where excess rewards are allocated among the non-competitors as the allocated condition. The collectively efficient competition level in the unallocated condition occurs if the number of competitors equals the number of rewards. Then, no rewards are lost and nobody incurred the cost of entering the competition without being able to receive a reward. The collectively efficient competition level in the allocated condition occurs if nobody competes. Then, nobody incurs an unnecessary cost and the rewards are allocated regardless. Hence, if people would condition their competition level on whether competing is socially beneficial or socially wasteful, participants should compete less in the allocated condition than in the unallocated condition. We will compare how people compete in the allocated and unallocated condition and assess the consequences of their competition behavior in terms of collective (in)efficiency. To compare collective (in)efficiency between conditions, we will use a collective inefficiency score that indicates the proportion of members in the group that would need to change their competition decision in order to achieve the collectively efficient allocation of rewards.

On the one hand, we can expect actors to compete less in the allocated condition than in the unallocated condition. Self-interested actors may reason that the potential payoff of not competing is higher in the allocated than in the unallocated condition. Indeed, the difference between both conditions is that in the former one can obtain a free reward despite abstaining from competition whereas in the latter one cannot. The potential payoff of competing is similar in both conditions. Hence, the incentive to not compete will generally be higher in the allocated condition than in the unallocated condition (see Appendix Section A1 for a detailed comparison of the individual expected payoffs of competing and not competing in both conditions). Altruistically motivated actors may reason that competing in the allocated condition is always collectively inefficient and therefore harmful for the total group payoffs, because the costs of competition are not required to allocate the rewards. In contrast, competing is not collectively inefficient in the unallocated model as long as the number of competitors does not exceed the number of rewards. Hence, also altruistically motivated actors can be expected to generally compete less in the allocated condition than in the unallocated condition (see Appendix Section A2 for the total group payoffs by each possible allocation of rewards in both conditions).

On the other hand, there is the potential for actors to be in a social trap of excessive competition both in the allocated and unallocated condition. That is, people may compete because they believe others compete, regardless of whether this competition is socially beneficial or wasteful. If self-interested actors believe that there will be high levels of competition (i.e., more competitors than rewards) also in the allocated condition, they know that there will be no rewards for non-competitors. They can then only obtain a reward by competing, and may therefore compete despite it being socially wasteful. Altruistically motivated actors often condition their altruism on the altruism of others^17,18^. If they believe that many others will compete despite this being socially wasteful, they may be less likely to abstain from the competition as well. Hence, the beliefs on how many others will compete may be important in explaining whether people compete similarly or not in the allocated and unallocated condition.

Whether people compete less if allocating scarce goods via competing is socially wasteful instead of socially beneficial also has important practical implications. In areas of excessive competition, institutional reforms are sometimes suggested through which the need for competition is lowered. For example, academics sometimes compete for tenured positions through obtaining grants. Writing grant proposals takes a considerable effort, especially considering that only a small fraction of proposals can be funded. As a solution to reduce academic competition, it is sometimes suggested to no longer require academics to write proposals and rather allocate grants among them (semi)randomly^19^. Such reforms will only be successful if people indeed compete less if competition is not necessary to allocate rewards. For example, no longer requiring academics to compete through grant proposals would only work if they do not shift their competition to other areas such as increasingly competing via publication quantity.

In our experiment, we have 204 participants making competition decisions in groups of six. We study whether participants compete differently in the allocated and unallocated condition in six settings that vary in the cost–benefit ratio of competing and the number of rewards. First, we vary whether the cost–benefit ratio of entering the competition is low or high. With the low cost–benefit ratio, participants receive 20 monetary units (MU) that they can either keep or use to enter the competition for a reward of 80 MU. With the high cost–benefit ratio, participants receive 50 MU that they can either keep or use to enter the competition for a reward of 60 MU. Second, we vary the number of rewards between a low (1 reward for 6 actors), medium (3 rewards for 6 actors), and high number (5 rewards for 6 actors). The combination of the cost–benefit ratio (2 settings) and the number of rewards (3 settings), gives us a total of six settings (2 × 3) for both the allocated and unallocated condition. This gives a more robust overview of whether people compete differently under the allocated and unallocated condition than if we had only studied one specific set of costs and number of rewards. To examine the role of beliefs, we also asked participants to report on their beliefs on the number of other competitors before making their own decision on whether to compete.

A first analysis of the allocated condition showed that people compete more with lower competition costs and a higher number of rewards^14^. However, there has been no comparison between the allocated and unallocated condition yet, which is what we need in order to test whether people compete differently in situations where competition is socially beneficial and socially wasteful. The unallocated versus allocated condition is varied between participants, the other six settings are varied within participants. This means that each participant makes a total of six competition decisions (one in each setting), with the groups being randomly reassigned after each decision such that participants compete against different people in each interaction. This repeated aspect of the competition (against different others) resembles to some extent the real-life competition examples provided earlier. For example, after having competed for a promotion, employees who lost may compete for subsequent promotions, and employees who won and are promoted will eventually also enter competitions again for yet further promotion. The same holds for job vacancies; even after having filled a vacancy, people may want to compete for new vacancies when they want to switch jobs. Competing for grants is also an example where academics have to compete repeatedly, as each grant typically only provides funding for a few years, after which new funding has to be sought. More details on the experiment can be found in the section “Materials and methods”.

## Results

**Figure 1:**
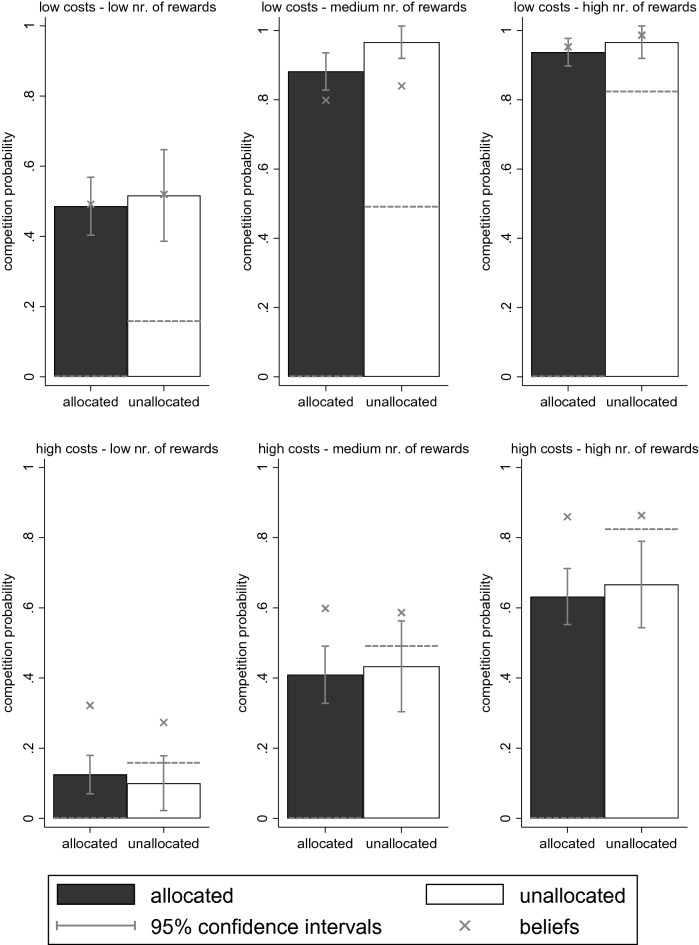
Competition in the allocated and unallocated condition. Mean competition proportions per condition and setting are shown with bars, including 95% confidence intervals with vertical spikes. Mean beliefs on the proportion of competitors are added with ‘x’ markers. The collectively efficient competition probabilities are added with horizontal dashed lines. We observe that mean competition and beliefs are very similar in the allocated and unallocated condition for all six settings.

Figure 1 shows the proportion of participants that competed in the allocated and unallocated condition in each of our six settings. We see that the competition rates are strikingly similar in both conditions. Participants do not compete less in the allocated condition than in the unallocated condition, even though competition is not required to allocate the rewards in the allocated condition. The difference within settings between the allocated and unallocated condition is always insignificant. Also when combining information from all experimental settings to estimate the overall difference in the competition rate between the allocated and unallocated condition, we find an insignificant difference (3%, *p* = 0.24, see Appendix Table A13). Using equivalence testing with the two-one-sided *t* test (TOST) procedure, we establish that the difference in the competition rate between conditions is statistically equivalent to a null-effect (see Appendix Table A13).

We also see in Fig. 1 that the beliefs on how many others will compete are very similar in both conditions. This suggests that beliefs may play a role in explaining the lack of a difference in competition rates between conditions, which we will examine later. We see that beliefs are not always correct, especially not if the costs to enter the competition are high (lower panels in Fig. 1). With high competition costs, participants’ beliefs on the number of other participants that will compete are higher than the actual number of competitors. To the extent that beliefs are involved in competition decisions (as we will examine later), lowering beliefs to prevent socially wasteful competition may thus be especially useful when competition costs are high. Participants overestimate the number of other competitors similarly in the allocated and unallocated condition. Altogether, we thus see that there is hardly any difference between the allocated and unallocated condition in the actual and believed competition rates.

Recall that the efficient allocation of rewards in the allocated condition occurs if nobody competes, as then nobody incurs competition costs and all rewards are allocated regardless. The efficient allocation of rewards in the unallocated condition occurs if the proportion of rewards is equal to the proportion of competitors, as rewards can only be earned by incurring the competition costs in this condition. Hence, the two conditions call for different competition behaviors to establish the efficient allocation of rewards. That people compete similarly in both conditions thus suggests that there will be inefficiency. By comparing the observed competition probabilities in Fig. 1 (bars) with the collectively efficient competition probabilities (dashed lines), we can see what behaviors lead to collective inefficiency. We see that the observed competition probability exceeds the collectively efficient competition probability in the unallocated condition when the cost–benefit ratio of competing is low (upper panels in Fig. 1). We see that the observed competition probability falls behind the collectively efficient competition probability in the unallocated condition when the cost–benefit ratio of competing is high (lower panels in Fig. 1). Hence, for the unallocated condition, there is too much competition if competition costs are low and too little competition when competition costs are high, causing some inefficiency in both cases. For the allocated condition, the collectively efficient competition probability is zero, and the observed competition probability is always above this level, meaning that there is collective inefficiency due to excess competition.

**Figure 2:**
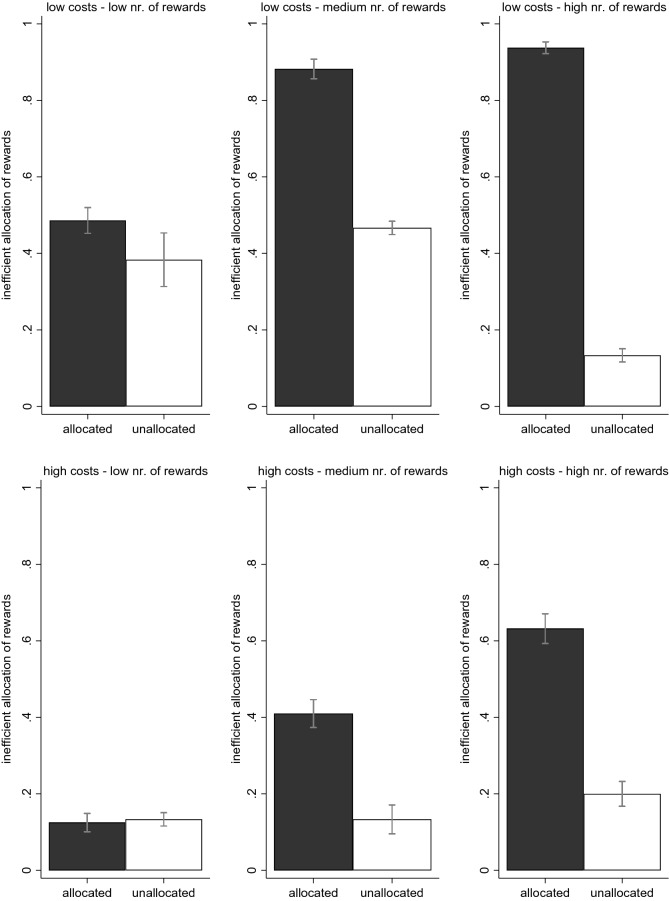
The observed efficiency of reward allocation by condition. For both conditions, the outcome variable is a collective inefficiency score that indicates the proportion of members in the group that would need to change their competition decision in order to achieve the collectively efficient allocation of rewards. Hence, the score has a theoretical minimum of 0 and maximum of 1. 95% confidence intervals are included. We observe higher inefficiency in the allocated condition than in the unallocated condition for 5 of the 6 settings.

In Fig. 2, we compare the collective (in)efficiency between the conditions by examining the percentage of members in the group that would need to change their competition decision in order to achieve the collectively efficient allocation of rewards. While Fig. 2 shows some inefficiency in both conditions, inefficiency is considerably and significantly higher in the allocated condition than in the unallocated condition in most settings. Whereas the inefficiency is always below 50% in the unallocated condition, it is above 50% in half of the settings in the allocated condition. The observed competition rates are thus relatively efficient for the unallocated condition and largely inefficient for the allocated condition. Note that a reverse pattern could in theory also have been possible. Had most participants abstained from competition in both conditions, then inefficiency would have been higher in the unallocated condition than in the allocated condition. Such inefficiently low competition in the unallocated condition occurred sometimes with high costs of competing (see also Fig. 1). However, our results largely suggest a tendency for too much instead of too little competition. Participants will compete for rewards regardless of whether this competition is necessary, causing large efficiency losses in situations where competition is not necessary (allocated condition).

To examine the role of beliefs in competition behavior, we estimate an individual fixed effect regression of participants’ competition decisions by their beliefs on the proportion of their group members that will compete. The individual fixed effect reduces confounding by between-participant confounders, and we also control for the returns of competing (the combination of the cost–benefit ratio of competing and number of rewards). Results are shown in Appendix Table A14. We find a strong and significant effect of beliefs on competition behavior. Each percentage point increase in one’s belief that others will compete increases one’s own competition by half a percentage point. We find this effect size both in the allocated and in the unallocated model. In addition, we estimate a regression model with a fixed effect for the experimental setting (the combination of the cost–benefit ratio of competing and number of rewards). In this regression, we examine the effect of beliefs regarding the proportion of one’s group members that will compete on one’s own decision to compete *within* experimental settings, thereby further reducing the confounding by the expected returns of competing. Results are shown in Appendix Table A15, and again show a considerable and significant positive effect of beliefs on competition behavior that is similar for both conditions. While there was already some indication that beliefs mattered for competition behavior in the allocated condition^14^, this was not previously tested and was limited only to the allocated condition. The similarly strong effect of beliefs in both conditions suggests that competition decisions are for a large part driven by beliefs on others’ competition regardless of whether competing is socially beneficial or socially wasteful.

In sum, we find a strong similarity in competition rates between the allocated and unallocated condition and a large association between beliefs of others’ competition and one’s own competition in both conditions. This coupled with the finding that these beliefs are similar in the allocated and unallocated condition suggests that beliefs indeed play a large role in the similarity in competition behavior in the allocated and unallocated condition, and hence in the inefficiently large competition in the allocated condition where competition is socially wasteful.

## Discussion

We find a striking similarity in competition rates in situations where competition is socially beneficial and socially wasteful. Whereas the observed competition level is relatively efficient when scarce goods can only be obtained through competition, it is largely inefficient when scarce goods could also have been obtained without competition. Hence, large efficiency gains are possible if we could lower competition in situations where goods can also be allocated without incurring the costs of competition. Our results suggest that beliefs on others’ competition behavior may be important to reduce socially wasteful competition. People compete more if they believe others will also compete, regardless of whether competing is socially wasteful. We find an indication of a social trap wherein everybody competes because they believe everybody else competes, even though most would have been better off by not competing. Interventions aimed at lowering beliefs on others’ competition may be an effective method of lowering competition in situations where competition is socially wasteful.

Lowering beliefs on how many others will compete may be more effective to reduce wasteful competition than lowering the need for competition. Our results suggest that people will compete even if competition is not necessary to allocate goods, at a rate similar to how they would compete if competition is necessary to allocate goods. This insight is especially important for attempts at reducing excessive competition in contemporary societies, where there are often many different and interlinked ways to compete. Lowering the need to compete in one area may simply shift the competition to another area. Instead, efforts to reduce beliefs that other people will compete in general may be more worthwhile.

We examined how competition behavior depends on whether competing is socially beneficial or socially wasteful using a simple competition experiment, in which the decision to compete is binary and the costs of competing are monetary. Future research is necessary to examine to what extent our findings generalize to other forms of competition. One way forward is to examine more gradual forms of competition. For example, individuals may choose their competition level within a continuous range, with rewards being allocated to those who selected the highest competition levels. Future research can also extend the analysis by examining competition not via monetary costs but rather via effort, for example using real effort tasks^20^. Moreover, it would be worthwhile to move the competition experiments outside of the lab to the real world^21^. Finally, because our sample largely consists of university undergraduates, future research is needed to assess to what extent our findings generalize to other samples such as older adults or populations with a different education level. With the ubiquity of human competition, further work on identifying the sources of wasteful competition may bring large efficiency gains on many fronts.

## Materials and methods

We conducted the experiment at the Experimental Laboratory for Sociology and Economics (ELSE) at Utrecht University in the winter of 2017/2018. The experiment was programmed with z-Tree software^22^. Participants were recruited amongst students at Utrecht University using the internet recruitment system ORSEE^23^. We divided a total of 204 participants into two conditions: 60 in the unallocated condition and 144 in the allocated condition. Previous research suggests that for the unallocated condition sufficient power is obtained with about 60 participants^13^. We did not have comparable information for the allocated condition and therefore recruited more participants for this condition. Within each condition, participants played the competition game under several settings that varied in the costs of competing and the number of rewards. Participants received on average 9 euros (min = 5, max = 15) and average experiment duration was 40 min. The amount they earned depended on their decisions and chance. Virtually all participants were students at Utrecht University, 84 were Dutch, and 120 were from various other countries. Participants were on average 24 years old, 144 were female, 59 were male, and one of another gender.

We think it is unlikely that our results can be explained by our sample being predisposed to competition at all costs. First, our sample consists for a large part of females (70%), and research suggests that females have relatively low tendencies to compete compared to males^24^. Second, we have a relatively diverse sample in terms of national background compared to other experiments (with 120 out of 204 participants being from various different countries other than the Netherlands), making it unlikely that our results are driven by adverse selection in terms of national culture of competitiveness^2^. Third, although our sample consists of mostly younger people, research shows that younger people do not compete more than older adults^25,26^. Fourth, our observed competition rates are similar to the competition rates found in a prior experiment that used the same competition model (using the unallocated condition) but with a different sample in another country (Switzerland) and using somewhat different written instructions^13^.

Participants took part in one of ten sessions (7 allocated, 3 unallocated). They were randomly placed in an individual cubicle, so they could not see, or communicate with, each other. They were informed through written instructions that they would play a competition game in groups of six, that there were six rounds of the competition game in total, and that the groups would be randomly reassigned every round. After reading the instructions, participants were given 5 questions to test their understanding of the game. Upon completion, they were shown which questions they had answered correctly. 183 participants had all answers correct, 17 participants had 4 answers correct, and 4 participants had 3 or 2 answers correct. One trial round of the game was played after these questions to get familiar with the game. After the experiment, participants were asked to report their understanding of the experiment. 195 reported a good understanding, 9 reported partial understanding, and nobody reported no understanding. Throughout the experiment, participants had the opportunity to ask questions to the lab official, but rarely made use of this opportunity.

We took multiple steps to ensure that participants understood whether and how excess rewards were allocated or unallocated. First, instructions explicitly mentioned whether excess rewards would be allocated among non-competitors (allocated condition) or not (unallocated condition). Second, the printed instructions were followed by three example scenarios, one of which included the scenario where 1 of the 6 actors compete for 3 rewards. Participants in the allocated condition were told that in this example scenario, 1 reward would go to the competitor and the 2 other rewards would go to 2 non-competitors. Participants in the unallocated condition were told that in this example scenario, only the 1 competitor would get the reward and the 5 non-competitors would not get a reward. Third, we tested participants’ understanding of whether excess rewards are allocated or unallocated with one of the questions in the pre-experiment quiz (“What happens if there are more rewards than competitors?”). This question was answered correctly by 95% of the participants (98% in the unallocated condition and 94% in the allocated condition). Finally, after each decision, the possible individual outcomes were shown. In the unallocated condition the three possible outcomes shown were: (1) competing and no reward, (2) competing and a reward, and (3) not competing and no reward. In the allocated condition, a fourth possible outcome was shown, namely not competing and obtaining a reward. Hence, after each decision, participants were also reminded whether winning a reward without competing was a possibility or not. The instructions, example scenarios, and test questions are available in the Appendix. The data and analysis scripts are openly available at 10.17605/OSF.IO/BY7WP.

A prior publication has analyzed the absolute level of competition in the allocated condition to study relative deprivation^14^. However, there has been no comparison of the unallocated and allocated condition yet. In the current study, we are interested in comparing the level of competition and collective efficiency between the allocated and unallocated condition. Within each condition, we varied the number of rewards and the cost–benefit ratio of competing across the six rounds. The number of rewards varied across three values: low (1), medium (3), and high (5). The cost–benefit ratio of competing varied between a low and high value. For the low cost–benefit ratio, participants received 20 points that they could either keep or use to enter the competition for a reward of 80 points. For the high cost–benefit ratio, participants received 50 points that they could either keep or use to enter the competition for a reward of 60 points. The exchange rate was 35 points for 1 euro. The combination of the three possible numbers of rewards and two possible competition costs leads to six possible settings in each condition, and each participant made a competition decision in each setting once. This allows us to compare the level of competition and collective efficiency between the allocated and unallocated condition for six settings varying in the number of rewards and competition costs.

Every participant played three consecutive rounds with high competition costs and three consecutive rounds with low competition costs. It was randomly varied across sessions whether the participants began with low or high competition costs. The order of low, medium, and high number of rewards was also randomly varied across sessions. We did not show participants the total points they had earned over prior rounds to diminish prior outcomes affecting decisions in subsequent rounds. Informed consent was obtained from all participants and the experimental procedures were approved by the Ethics Committee of the Faculty of Social and Behavioral Sciences of Utrecht University. All methods were performed in accordance with the relevant guidelines and regulations.

## Supplementary Information

Supplementary Information.

## Data Availability

All data are openly available at 10.17605/OSF.IO/BY7WP. Questions can be directed to k.d.otten@uu.nl.
