# Supplementary material for: Human competition is not lower if competing is socially wasteful instead of socially beneficial
Source: Sci Rep. 2022 Jun 24;12:10740. doi: 10.1038/s41598-022-14891-7 (PMC9232650; doi:10.1038/s41598-022-14891-7)
Supplement: Supplementary file 1 — Supplementary Information. [file 41598_2022_14891_MOESM1_ESM.pdf]

Appendix for:

Human competition is not lower if competing is socially wasteful instead of socially beneficial

Kasper Otten[1,*]

[1] Utrecht University, Department of Sociology

[*] Corresponding author: Padualaan 14, 3584 CH Utrecht, the Netherlands; k.d.otten@uu.nl; +31 30 253 8813

Table of contents

A1. Individual payoffs

A2. Total group payoffs

A3. Statistical analyses

A4. Experimental instructions and test questions

A1. Individual payoffs

In this section, we present the actor's expected payoff for competing and not competing under all possible numbers of other competitors (n – 1) for all six examined settings (low cost-benefit ratio with 1, 3, or 5 rewards and high cost-benefit ratio with 1, 3, or 5 rewards; group size is always 6) for both conditions. In the low cost-benefit ratio settings, participants receive an endowment of 20 monetary units (MU) that they can use to enter the competition or to keep if they do not enter the competition. A reward is worth 80 MU in the low cost-benefit ratio setting. Table A1(a) shows the expected payoff of competing and not competing in the setting with the low cost-benefit ratio and 1 reward in the unallocated condition. We see that the expected payoff of competing depends on the number of other competitors. If there are no other competitors, the actor can be sure of a reward when competing, and therefore obtains an expected payoff of 80 MU. If there is one other competitor, there is a one-half probability of coming out a winner and a one-half probability of coming out a loser when competing, giving an expected payoff of ½ × 80 + ½ × 0 = 40 MU. If there are 2 other competitors, there is a one-third probability of coming out a winner and a two-thirds probability of coming out a loser, giving an expected payoff of 1/3 × 80 + 2/3 × 0 = 26.7 MU, and so on. The expected payoff of not competing is always 20 MU in the unallocated condition. From Table A1(a), we can see that there is no dominant strategy in the setting with the low cost-benefit ratio and 1 reward for the unallocated condition, i.e., there is no strategy that gives the highest expected payoff regardless of what others do. An individual actor would obtain the highest expected payoff by competing instead of not competing if the number of other competitors does not exceed 3. However, if the number of other competitors does exceed 3, an individual actor obtains the highest expected payoff by not competing instead of competing.

Table A1(b) shows the expected payoff of competing and not competing for the same setting with the low-cost benefit ratio and 1 reward, but in the allocated condition. The expected payoff of competing is the same in the unallocated and allocated condition, but the expected payoff of not competing is higher in the allocated condition. In particular, when the number of other competitors is 0, there is a possibility that the actor gets the 1 reward without competing. This means the actor would save the costs of competing (20 MU) and potentially obtain the reward (80 MU) with some probability. The probability of obtaining the 1 reward if nobody competes is 1/6, giving a total expected payoff of not competing of 20 + 1/6 × 80 = 33.3 MU. When there is somebody else that competes (number of other competitors ≥ 1), the 1 reward will go to the

competitor(s) and the expected payoff of not competing is therefore only 20 MU, as in the unallocated condition.

Table A2(a) shows the expected payoff of competing and not competing in the setting with the low cost-benefit ratio and 3 rewards in the unallocated condition. If there are fewer than 2 other competitors, the actor can be sure of a reward when competing, and therefore obtains an expected payoff of 80 MU. If there are 3 other competitors, there is a probability of ¾ of winning and ¼ of losing when competing, giving an expected payoff of ¾ × 80 + ¼ × 0 = 60 MU. If there are 4 other competitors, there is a 3/5 probability of winning and 2/5 of losing when competing, giving an expected payoff of 3/5 × 80 + 2/5 × 0 = 48 MU, and so on. In Table 2(b) we show the expected payoffs for the allocated condition, where it is possible to save the costs of competing (20 MU) and potentially obtain a reward (80 MU) with some probability. The probability of obtaining one of the rewards when not competing and when there are also no other competitors is 3/6, giving an expected payoff of not competing of 20 + 3/6 × 80 = 60 MU. When there is one other competitor, there are only 2 rewards left for non-competitors, giving an expected payoff of not competing of 20 + 2/6 × 80 = 46.7, and so on. Tables A3-A6 show the expected payoffs of competing and not competing for the other four settings (low cost-benefit ratio with 5 rewards, and high cost-benefit ratio with 1, 3, or 5 rewards). These expected payoffs are calculated with the same approach as described above. In the settings with a high cost-benefit ratio, participants receive an endowment of 50 monetary units (MU) that they can use to enter the competition or to keep if they do not enter the competition. A reward is worth 60 MU in the high cost-benefit ratio setting.

By comparing the expected payoffs of competing and not competing between the unallocated and allocated condition for each of the six settings, we can obtain an indication of the extent to which self-interested actors can be expected to compete differently in the unallocated and allocated setting. For **setting 1** (low cost-benefit ratio, 1 reward; Table A1), we see that there is no dominant strategy, neither in the unallocated condition nor in the allocated condition. However, the expected payoff of not competing is somewhat higher in the allocated condition, which could lead to lower competition probabilities in the allocated condition. For **setting 2** (low cost-benefit ratio, 3 rewards; Table A2), we see that there is a dominant strategy to compete, both in the unallocated and allocated condition. Hence, if we expect actors to make competition decisions purely based on their self-interest, we would not expect a difference between the unallocated and allocated condition here. For **setting 3** (low cost-benefit ratio, 5

rewards; Table A3), we see that there is a dominant strategy to compete in the unallocated condition, but no dominant strategy in the allocated condition, so we would expect lower competition probabilities in the allocated condition. For **setting 4** (high cost-benefit ratio, 1 reward; Table A4), we see that there is no dominant strategy in the unallocated condition and a (weakly) dominant strategy in the allocated condition to not compete. Hence, we would expect lower competition probabilities in the allocated condition. For **setting 5** (high cost-benefit ratio, 3 rewards; Table A5), we see that there is no dominant strategy in the unallocated condition and a (strongly) dominant strategy in the allocated condition to not compete. Hence, we would expect lower competition probabilities in the allocated condition. For **setting 6** (high cost-benefit ratio, 5 rewards; Table A6), we see that there is a (weakly) dominant strategy to compete in the unallocated condition and a (weakly) dominant strategy to not compete in the allocated condition. Hence, this is an especially interesting setting where we would expect direct opposite competition behaviors in the two settings. Altogether, we would expect competition to be lower in the allocated condition than in the unallocated condition for almost all settings (except for setting 2), and an especially large difference in setting 6. However, as shown in Figure 1 of the main text, competition rates are virtually similar in each setting and the difference between conditions is also insignificant when comparing within settings (analysis reported in Table A13).

Table A1. Individual payoffs for setting with the low cost-benefit ratio and 1 reward

(a) Unallocated condition

	Number of other competitors (n – 1)					
	0	1	2	3	4	5
Compete	80	40	26.7	20	16	13.3
Do not compete	20	20	20	20	20	20

(b) Allocated condition

	Number of other competitors (n – 1)					
	0	1	2	3	4	5
Compete	80	40	26.7	20	16	13.3
Do not compete	33.3	20	20	20	20	20

Table A2. Individual payoffs for setting with the low cost-benefit ratio and 3 rewards

(a) Unallocated condition

	Number of other competitors (n – 1)					
	0	1	2	3	4	5
Compete	80	80	80	60	48	40
Do not compete	20	20	20	20	20	20

(b) Allocated condition

	Number of other competitors (n – 1)					
	0	1	2	3	4	5
Compete	80	80	80	60	48	40
Do not compete	60	46.7	33.3	20	20	20

Table A3. Individual payoffs for setting with the low cost-benefit ratio and 5 rewards

(a) Unallocated condition

Number of other competitors (n – 1)

	0	1	2	3	4	5
Compete	80	80	80	80	80	66.7
Do not compete	20	20	20	20	20	20

(b) Allocated condition

Number of other competitors (n – 1)

	0	1	2	3	4	5
Compete	80	80	80	80	80	66.7
Do not compete	86.7	73.3	60	46.7	33.3	20

Table A4. Individual payoffs for setting with the high cost-benefit ratio and 1 reward

(a) Unallocated condition

Number of other competitors (n – 1)

	0	1	2	3	4	5
Compete	60	30	20	15	12	10
Do not compete	50	50	50	50	50	50

(b) Allocated condition

Number of other competitors (n – 1)

	0	1	2	3	4	5
Compete	60	30	20	15	12	10
Do not compete	60	50	50	50	50	50

Table A5. Individual payoffs for setting with the high cost-benefit ratio and 3 rewards

(a) Unallocated condition

Number of other competitors (*n* – 1)

	0	1	2	3	4	5
Compete	60	60	60	45	36	30
Do not compete	50	50	50	50	50	50

(b) Allocated condition

Number of other competitors (*n* – 1)

	0	1	2	3	4	5
Compete	60	60	60	45	36	30
Do not compete	80	70	60	50	50	50

Table A6. Individual payoffs for setting with the high cost-benefit ratio and 5 rewards

(a) Unallocated condition

Number of other competitors (*n* – 1)

	0	1	2	3	4	5
Compete	60	60	60	60	60	50
Do not compete	50	50	50	50	50	50

(b) Allocated condition

Number of other competitors (*n* – 1)

	0	1	2	3	4	5
Compete	60	60	60	60	60	50
Do not compete	100	90	80	70	60	50

A2. Total group payoffs

In this section, we present the total group payoffs for each possible number of competitors per setting in both the unallocated and allocated condition. Table A7(a) shows the total group payoffs for each potential number of competitors in the setting with the low cost-benefit ratio and 1 reward in the unallocated condition. Recall that participants in this setting receive an endowment of 20 MU that they can use to enter the competition and that a reward is worth 80 MU. If there are no competitors, all six actors keep their endowment of 20 MU, giving a total group payoff of 6 × 20 = 120 MU. If 1 of the 6 actors competes, that actor obtains the reward of 80 MU and the other 5 actors keep their endowment of 20 MU, giving a total group payoff of 80 + 5 × 20 = 180 MU. If 2 of the 6 actors compete, 1 actor obtains the reward of 80 MU, 1 actor spent the endowment to enter the competition but loses, and 4 actors keep their endowment of 20 MU, giving a total group payoff of 80 + 0 + 4 × 20 = 160 MU, and so on.

Table A7(b) shows the total group payoffs for the same setting but in the allocated condition. If there are no competitors, the 1 reward of 80 MU gets randomly allocated to one of the six actors. This means that one actor gets 80 MU on top of the endowment of 20 MU, for a combined payoff of 100 MU. The other 5 actors that also did not compete get to keep their endowment of 20 MU. The total group payoffs are then 100 + 5 × 20 = 200 MU. If 1 of the 6 actors competes, only the actor that competed obtains the reward of 80 MU and the other 5 actors keep their endowment of 20 MU, giving a total group payoff of 80 + 5 × 20 = 180 MU, and so on. Tables A7-A12 show the total group payoffs in all six settings and both conditions for each possible number of competitors. The number of competitors that leads to the highest total group payoff is highlighted in green. We see that for the unallocated condition, total group payoffs are always highest when the number of competitors equals the number of rewards. We see that for the allocated condition, total group payoffs are always highest when nobody competes. Hence, actors who would want to maximize total group payoffs would compete less in the allocated condition than in the unallocated condition.

Table A7. Total group payoffs for setting with the low cost-benefit ratio and 1 reward

(a) Unallocated condition

Total number of competitors	0	1	2	3	4	5	6
Total payoffs	120	180	160	140	120	100	80

(b) Allocated condition

Total number of competitors	0	1	2	3	4	5	6
Total payoffs	200	180	160	140	120	100	80

Table A8. Total group payoffs for setting with the low cost-benefit ratio and 3 rewards

(a) Unallocated condition

Total number of competitors	0	1	2	3	4	5	6
Total payoffs	120	180	240	300	280	260	240

(b) Allocated condition

Total number of competitors	0	1	2	3	4	5	6
Total payoffs	360	340	320	300	280	260	240

Table A9. Total group payoffs for setting with the low cost-benefit ratio and 5 rewards

(a) Unallocated condition

Total number of competitors	0	1	2	3	4	5	6
Total payoffs	120	180	240	300	360	420	400

(b) Allocated condition

Total number of competitors	0	1	2	3	4	5	6
Total payoffs	520	500	480	460	440	420	400

Table A10. Total group payoffs for setting with the high cost-benefit ratio and 1 reward

(a) Unallocated condition

Total number of competitors	0	1	2	3	4	5	6
Total payoffs	300	310	260	210	160	110	60

(b) Allocated condition

Total number of competitors	0	1	2	3	4	5	6
Total payoffs	360	310	260	210	160	110	60

Table A11. Total group payoffs for setting with the high cost-benefit ratio and 3 rewards

(a) Unallocated condition

Total number of competitors	0	1	2	3	4	5	6
Total payoffs	300	310	320	330	280	230	180

(b) Allocated condition

Total number of competitors	0	1	2	3	4	5	6
Total payoffs	480	430	380	330	280	230	180

Table A12. Total group payoffs for setting with the high cost-benefit ratio and 5 rewards

(a) Unallocated condition

Total number of competitors	0	1	2	3	4	5	6
Total payoffs	300	310	320	330	340	350	300

(b) Allocated condition

Total number of competitors	0	1	2	3	4	5	6
Total payoffs	600	550	500	450	400	350	300

A3. Statistical analyses

Table A13. Regression model of competition probability with setting fixed effects

		90% confidence interval	
	Estimate	Lower bound	Upper bound
Experimental condition	0.03	-0.01	0.07
	(0.03)		
Intercept	0.58***	0.56	0.60
	(0.01)		
N observations	1224		
R^2 (overall)	0.00		

Note: * *p* < 0.05, ** *p* < 0.01, *** *p* < 0.001. To get a clean comparison between the unallocated and allocated (ref.) condition unconfounded by the experimental setting (costs of competing and number of rewards), we employ setting fixed effects. Standard errors in parentheses. To statistically test whether the difference between the allocated and unallocated condition is equivalent to zero, we use equivalence testing with the two-one-sided t-test (TOST) procedure. With this procedure, a smallest effect size of interest is chosen and then used to obtain an equivalence range. The upper bound of this range is obtained by adding the smallest effect size of interest to one's prediction, the lower bound of the range is obtained by subtracting the smallest effect size of interest from one's prediction. If the 90% confidence interval of the observed effect falls within the equivalence range, there is significant equivalence. In line with prior research on the model[14], we regard a difference of 7.5% as the smallest effect size of interest. This means that the equivalence range of the null-effect is [-7.5%, 7.5%]. The observed 90% confidence interval of [-1%, 7%] falls within this equivalence range, signifying statistical equivalence of a null-effect.

Table A14. Regression model of competition probability by beliefs with individual fixed effects

belief on proportion of other competitors	0.53***	0.54***
	(0.06)	(0.07)
belief on proportion of other competitors × unallocated condition		-0.04
		(0.08)
low rewards - high costs	-0.28***	-0.28***
	(0.04)	(0.04)
medium rewards - low costs	0.25***	0.25***
	(0.04)	(0.04)
medium rewards - high costs	-0.13***	-0.13***
	(0.04)	(0.04)
high rewards - low costs	0.21***	0.21***
	(0.05)	(0.05)
high rewards - high costs	-0.04	-0.04
	(0.04)	(0.04)
Intercept	0.23***	0.23***
	(0.04)	(0.04)
N observations	1224	1224
R^2 (within)	0.44	0.44

Note: * *p* < 0.05, ** *p* < 0.01, *** *p* < 0.001. Regression with participant fixed effects. Standard errors in parentheses. Reference category for the condition is the allocated condition. Reference category for the settings is the low rewards – low costs setting.

Table A15. Regression model of competition probability by beliefs with setting fixed effects

belief on proportion of other competitors	0.56***	0.57***
	(0.06)	(0.06)
unallocated condition		0.06
		(0.06)
belief on proportion of other competitors × unallocated condition		-0.06
		(0.08)
Intercept	0.21***	0.19***
	(0.04)	(0.04)
N observations	1224	1224
R^2 (within)	0.08	0.08

Note: * $p < 0.05$, ** $p < 0.01$, *** $p < 0.001$. Regression with setting fixed effects (cost-benefit ratio of competing × number of rewards). Standard errors in parentheses. Reference category for the condition is the allocated condition.

A4. Experimental instructions and test questions

Note: these are the instructions for the allocated setting. The instructions for the unallocated setting are largely similar. The differences in text between settings are described through square brackets.

Instructions

Welcome to this experiment and thank you for coming. Please read the following instructions carefully. These instructions are the same for all participants. The instructions state everything you need to know in order to participate in the experiment. If you have any questions, please raise your hand. One of the experimenters will approach you in order to answer your question.

You can earn money by means of earning Game Points (GP) during the experiment. The number of GP that you earn depends on your own choices. At the end of the experiment, the total number of GP that you earned during the experiment will be exchanged at the rate of:

35 GP = 1 Euro

The money you earn will be rounded off to 50 cents at the end of the experiment and will be paid out in cash, without other participants being able to see how much you earned. During the experiment you are not allowed to communicate with other participants. Please turn off your mobile phone and put it in your bag. Also, you may only use the functions on the screen that are necessary to carry out the experiment. Thank you very much.

Overview of the Session

The experiment consists of six rounds and will last about 45 minutes. First, there will be a practice round in which you can get experience with how your earnings are calculated. Then, we will turn to "The Experiment", during which you can actually earn Game Points (GP). Finally, you will be asked to fill in a questionnaire. While you do so, your earnings will be counted. Please remain seated after having filled in the questionnaire until the payment has taken place. All choices and information you provide will remain anonymous.

Because you play together with other persons, you will sometimes have to wait until the other persons have made their decision. These waiting times are incorporated in the total duration of the experiment.

Overview of the Experiment

In this experiment, you will play a competition game in groups of six. There are six rounds of the competition game in total, and **every round you will be randomly assigned to a new group.**

An example screen of the competition game is shown in Figure A1.

Figure A1. Example screen of competition game

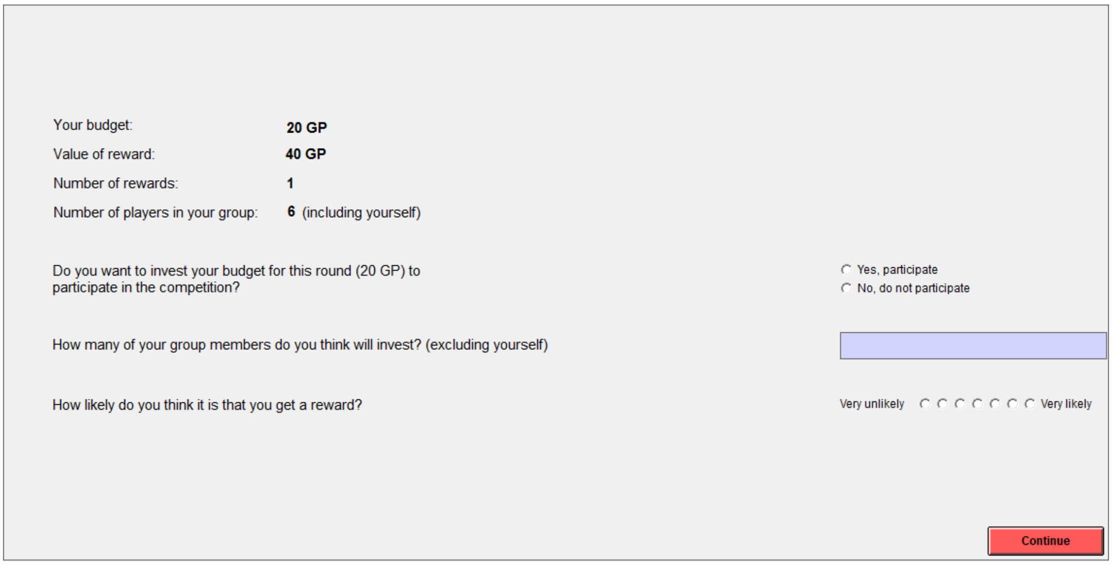

The rules of the competition game are as follows:

1. There are a number of rewards to be allocated among your group (1 in the example screen). You receive a budget (20 GP in the example screen) that you can invest to participate in the competition for these rewards. You can also choose to keep your budget and not participate in the competition.

2. The rewards will first be allocated to the persons who have participated in the competition (the competitors):

- If *the number of competitors exactly equals the number of rewards*, all competitors will get a reward and all persons that did not participate in the competition (the non-competitors) do not get a reward.
- If there are *more competitors than rewards*, not every competitor can get a reward. The rewards will be randomly allocated over the competitors. The rest of the competitors will not receive a reward, despite having spent their budget to participate in the competition. All non-competitors will not get a reward.

- If there are *fewer competitors than rewards,* there will be rewards left after all competitors have received a reward. Some of the non-competitors will therefore be randomly chosen to get one of the excess rewards. The rest of the non-competitors will not get a reward. [*Unallocated condition: "If there are fewer competitors than rewards, there will be rewards left after all competitors have received a reward. These rewards are not allocated. All non-competitors will not get a reward."*]

3. Before you choose to invest your budget in order to participate in the competition or to keep your budget and not participate, you will receive information about the value of the budget (20 GP in the example), the value of the rewards (40 GP in the example), and the number of rewards (1 in the example). You also see that the number of players in your group is always 6.

4. You must then decide whether or not to participate in the competition. If you decide to participate, you will automatically invest your budget. If you do not participate, you keep your budget. You will also be asked to indicate how many others you think will invest and how likely you think it is that you get a reward.

5. After all the members of your group have made their decisions, the rewards will be allocated. You will receive information about your outcome and the outcome of the other persons within your group.

6. You will be asked, among other things, to rate your satisfaction with your outcome compared to your group members.

7. You will be assigned a random new group, and a new round will start.

Thus, there are four possible outcomes for you in each round of the game based on your decision to compete, and those of the other group members:

1. Reward after participating. You do not keep your budget, but get the value of the reward (40 GP in the example).

2. No reward after participating. You do not keep the budget and get no reward (0 GP).

3. No reward after not participating. You keep the budget but get no reward (20 GP in the example).

4. Reward after not participating. You keep the budget and get a reward (20 + 40 = 60 GP in the example). [*This possibility is not given in the unallocated condition*].

Some Examples of the Game

Example 1 (fictive numbers):

If in a given competition there are 2 rewards, and 5 out of the 6 persons of a given group choose to be a competitor, then:

- the 1 non-competitor gets no reward, but keeps his/her budget;
- 2 of the 5 competitors get a reward, but do not keep their budget; and
- 3 of the 5 competitors get no reward, and lose their budget.

Example 2 (fictive numbers):

If in a given competition there are 3 rewards, and 1 out of the 6 persons of a given group chooses to be a competitor, then

<u>Outcome provided for the allocated condition:</u>

- the 1 competitor gets a reward, but does not keep his/her budget;
- 3 out of the 5 non-competitors get no reward, but keep their budget; and
- 2 of the 5 non-competitors get a reward, in addition to their initial budget.

<u>Outcome provided for the unallocated condition:</u>

- the 1 competitor gets a reward, but does not keep his/her budget;
- The 5 non-competitors get no reward, but keep their budget.

Example 3 (fictive numbers):

If in a given competition there is 1 reward, and 1 out of the 6 persons of a given group chooses to be a competitor, then:

- the 5 non-competitors get no reward, but keep their budget; and
- the 1 competitor gets the reward, but does not keep his/her budget.

End of Experiment

After you have finished the questionnaire at the end of the experiment, the experimenter will approach as quickly as possible to hand over the money you earned in the experiment. Other participants are not able to see how much you have earned. If you have any questions, please raise your hand and the experimenter will approach. Thank you very much for participating in this experiment.

Test Questions

1. With how many others are you in a group?
   a) 5 (correct)
   b) 7
   c) 9

2. Which one of the following three statements are correct?
   a) It does not cost me any points to participate in the competition
   b) I have to participate in the competition
   c) It is up to me whether or not I participate in the competition (correct)

3. What happens if there are fewer rewards than competitors?
   a) The rewards are randomly allocated over the competitors (correct)
   b) All competitors get a reward
   c) All competitors and some non-competitors get a reward

4. Which one of the following three statements are correct?
   a) My group will consist of the same people throughout all rounds of the experiment
   b) I will be randomly assigned a new group every round (correct)
   c) The groups will be changed only after the first three rounds

5. What happens if there are more rewards than competitors?

   <u>Answer categories for the allocated condition:</u>
   a) Only the competitors get a reward
   b) All competitors get a reward, and the rewards that are left are randomly allocated over the non-competitors (correct)
   c) All non-competitors get a reward

   <u>Answer categories for the unallocated condition:</u>
   a) All competitors get a reward, and the rewards that are left are not allocated (correct)
   b) All competitors get a reward, and the rewards that are left are randomly allocated over the non-competitors
   c) All non-competitors get a reward
